# Skin Anti-Aging Efficacy of Enzyme-Treated Supercritical Caviar Extract: A Randomized, Double-Blind, Placebo-Controlled Clinical Trial

**DOI:** 10.3390/nu16010137

**Published:** 2023-12-30

**Authors:** Gwi Hwan Na, SukJin Kim, Hyun Mook Jung, Sang Hun Han, Jehee Han, Yean Kyoung Koo

**Affiliations:** 1 Department of R&I Center, COSMAXBIO, Seongnam 13486, Republic of Korea; ghna@cosmax.com (G.H.N.); sukjin.kim@cosmax.com (S.K.); hyunmookjung@cosmax.com (H.M.J.); 2 Almas Caviar, Hwaseoung-si 18553, Republic of Korea; kredward@hanmail.net (S.H.H.); jeheehan5@gmail.com (J.H.)

**Keywords:** caviar, skin anti-aging, skin wrinkles, skin hydration, skin elasticity, skin whitening

## Abstract

Oxidative stress in the skin, induced by an unhealthy lifestyle and exposure to UVB radiation, leads to skin aging, including reduced elasticity, formation of wrinkles, moisture loss, and inflammation. In a previous study, we revealed the photoaging effects of enzyme-treated caviar extract (CV) by regulating collagen and hyaluronic acid synthase, melanogenesis, anti-oxidant mechanisms, and inflammation in a UVB irradiation-induced mice model. HPLC and MALDI-TOF were performed to determine the effect of enzyme treatment on the free amino acid contents and peptide molecular weight in supercritical caviar extract. As results of the analysis, CV is mainly composed of low-molecular-weight peptides consisting of leucine, tyrosine, and phenylalanine. Based on our in vitro and in vivo study, we conducted a clinical trial to assess the skin anti-aging efficacy of CV. In this randomized, double-blind, placebo-controlled trial, we measured indicators related to elasticity, wrinkles, and skin hydration at 4 and 8 weeks after consumption of CV. The subjects were categorized into caviar, combination, and placebo groups. After 4 weeks, skin hydration, dermal hydration, and transepidermal water loss all showed significant improvement. Furthermore, after 8 weeks, skin elasticity indexes—R2 (total elasticity), R5 (net elasticity), and R7 (ratio of elastic recovery to total deformation)—exhibited significant increases. Improvement in wrinkle indicators (Rmax, Ra, and Rz) and the whitening indicator melanin pigment was also observed. This is the first report showing that CV has significant skin anti-aging efficacy on human skin. In conclusion, our study suggests that CV can be used as skin anti-aging nutraceuticals through positive effects on skin condition in clinical trials.

## 1. Introduction

Caviar, the processed or salted roe of the sturgeon, is a rich source of amino acids, fatty acids, and minerals [1]. The omega-3 fatty acids and selenium present in caviar offer health benefits, including immune enhancement and support for male fertility [2,3]. In a recent in vitro study, docosahexaenoic acid (DHA) in caviar extracts stimulated adipocytes, promoting the production of adiponectin. This facilitates wound healing, anti-inflammatory, and collagen synthesis in the skin [4]. A multifunctional cosmetic incorporating caviar extract was shown to improve in the appearance of photoaged skin after 12 weeks of twice-daily use [5]. Caviar is widely used as a cosmetic to improve skin, but its clinical efficacy as a nutraceutical has not been proven.

Skin, a crucial organ comprising diverse cell types and multiple layers, functions as a physical barrier against environmental factors, regulates hydration levels, and maintains physiological balance [6,7]. The skin aging process involves both intrinsic and extrinsic factors [8]. Intrinsic aging is a natural progression encompassing chronological, natural, and biological aging, while extrinsic aging, such as exposure to sunlight, smoking, and malnutrition, contribute to the deterioration of the skin’s structure and function [9,10]. As aging progresses, essential components like elastin fibers, collagen, and other substances crucial for preserving skin health, radiance, and elasticity undergo degradation. The generation of reactive oxygen species (ROS) due to UVB exposure triggers the release of inflammatory cytokines and promotes melanin production, leading to skin dryness, reduced elasticity, pigment irregularities, and potential skin tumors [11,12]. Furthermore, ROS adversely affects collagen, ceramides, and hyaluronic acid—essential elements that fortify the skin barrier and retain moisture [13,14]. Consequently, in order to combat skin aging, it is imperative to mitigate ROS-induced melanin synthesis and prevent the degradation of hyaluronic acid, ceramides, and collagen.

**Figure 1:**
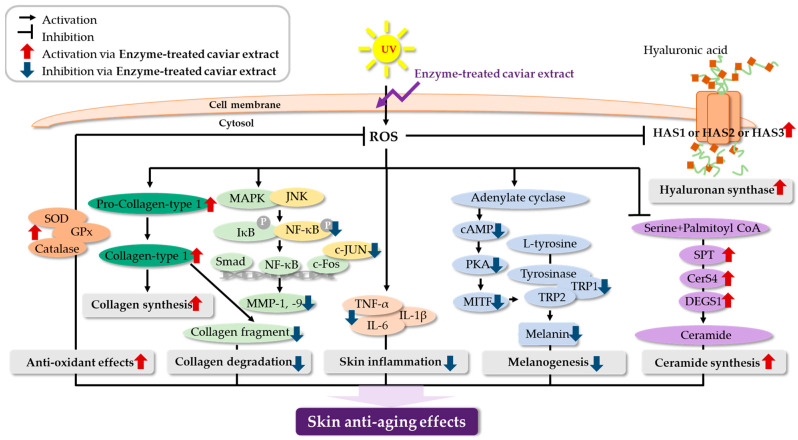
Mechanism of skin anti-aging efficacy of enzyme-treated caviar extract.

As shown in Figure 1, our previous study showed that enzyme-treated supercritical caviar extract (CV) prevents skin photoaging by improving skin wrinkles, elasticity, and whitening through various mechanisms, including antioxidant and anti-inflammatory pathways and collagen synthesis [15,16]. CV exhibited antioxidant efficacy by increasing enzyme activities such as SOD, catalase, and GPx. CV showed skin anti-aging efficacy by increasing collagen, ceramide, and hyaluronic acid synthesis by regulating gene expression such as collagen type-1, HAS, SPT, DERS4, and DEGS. Furthermore, CV not only suppressed collagen degradation by regulating MAPK signaling proteins, but also inhibited melanogenesis. 

Consequently, the current study aims to conduct a clinical evaluation of the safety and skin anti-aging efficacy of CV based on an in vivo study. For this purpose, we conducted a randomized, placebo-controlled clinical trial involving 45 adults aged 30 to 65 years with wrinkles and dry skin. CV was prescribed to two treatment groups (10 and 500 mg) over an 8-week period to assess its skin anti-aging efficacy by measuring parameters such as hydration, elasticity, melanin content, wrinkles, and transepidermal water loss (TEWL). The safety profile of CV was evaluated through hematological, biochemical, and urine tests conducted after the 8-week trial period. Furthermore, amino acid and protein analysis was performed to determine which components of CV exhibit skin anti-aging effects. Our study is the first report verifying the exceptional skin anti-aging efficacy of CV.

## 2. Materials and Methods

### 2.1. Preparation of CV

The supercritical extract and enzyme-treated extract of Beluga (Huso Huso) caviar used in this study was sourced from Almas Caviar Inc. (Gyeonggi-do, Republic of Korea). Almas Caviar Inc. is actively engaged in environmental conservation efforts aimed at preserving sturgeon resources (a CITES-listed item), the primary source of caviar. Their commitment involves employing Sustainable Technology, specifically species conservation science, in the caviar production process. Prozyme1000L, an endopeptidase obtained from a culture of the genus Bacillus, was used for enzymatic treatment of the caviar extract, and CV was prepared as previously described [15,16].

### 2.2. Free Amino Acid and Peptide Analysis

The high-performance liquid chromatography (HPLC) was used to determine free amino acid analysis of CV. Weigh 1 g of powder, add 10 times the amount of distilled water, and heat to solidify, and the solution is filtered to obtain an aqueous layer. The residue obtained by concentrating the aqueous layer to dryness under reduced pressure was dissolved in 0.2 N sodium citrate buffer (pH 2.2) and used as a sample for HPLC. Agilent LC system (Agilent Technologies, Santa Clara, CA, USA) and Capcellpak UG120 with properties of (250 mm × 4.6 mm × 5 μm) was used. The mobile phases were: A, 40 mM NaH2PO4 (pH 7.8) and B ACN:MeOH:DW (45:45:10 *v*/*v*) and the elution gradient was: 0–31 min, 95% A; 34 min, 44% A; 38 min, 0% A. The flow rate was 1.5 mL/min, and the absorbance of detection was 262 and 338 nm. Column temperature was set to 40 °C and injection volume was 10 μL. 

Peptide molecular weights of CVs were measured in linear mode using a RapifleX MALDI-TOF mass spectrometer (Bruker, Billerica, MA, USA). Sample preparation and peptide analysis were performed by modifying method Lu et al. [17].

### 2.3. Study Design and Dose Determination

This clinical trial had a randomized, placebo-controlled, double-blind design. It was conducted according to the guidelines of the applicable Global Medical Research Center (Seoul, Republic of Korea) from 22 April 2022 to 30 September 2022. The study protocol was reviewed and approved by the Institutional Review Board of the Global Medical Research Center (Protocol No.: GMRC-22415-HK). This trial is registered with registered with the Clinical Research Information Service (CRIS, no. KCT0008242) in the Korea Disease Control and Prevention Agency supported by the Ministry of Health and Welfare. The study protocol followed CONSORT 2010 checklist (Supplementary Table S1).

Table 1 provides a breakdown of the detailed capsule formulation for the daily dose (900 mg). The caviar group received a daily dose of CV at 500 mg, while the combination group was administered a combination of CV (10 mg), fish collagen (500 mg), ascorbic acid (125 mg), hyaluronic acid (240 mg), and biotin (7.5 mg). The Placebo group was provided with maltodextrin alone at 500 mg. All groups ingested 300 mg/tablet once a day, three tablets at each administration. 

In our prior animal experiments, doses of 20 mg/kg/day, 50 mg/kg/day, and 100 mg/kg/day of CV were orally administered to assess its effects on skin wrinkles and skin hydration [15,16]. These amounts were converted to human equivalent doses of 98 mg/day, 245 mg/day, and 491 mg/day, respectively, based on a human body weight of 60 kg [18]. Consequently, clinical trials were set at 500 mg/day to assess the efficacy of CV at a maximized dose considered safe.

### 2.4. Study Participants and Schedule

**Figure 2:**
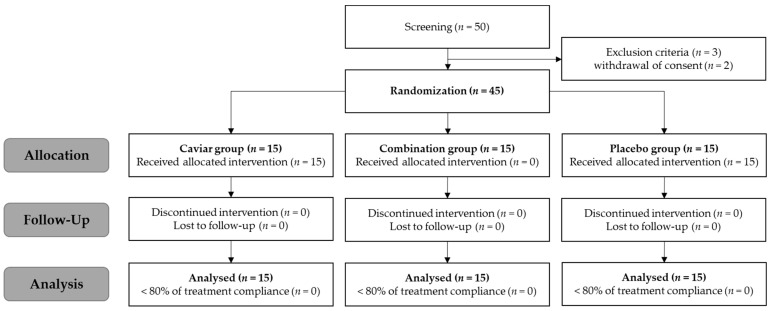
Consort flow diagram of enrolled participants in a randomized study evaluating enzymatically treated caviar extracts.

Healthy adults aged 30 to 60 years (*n* = 45) meeting the selection criteria and not falling under the exclusion criteria were recruited. Table 2 presents the personal information of the participants. Each subject underwent a demographic survey, medical history and drug administration history survey, clinical pathology test, pregnancy test (for women of childbearing age only), skin wrinkle evaluation, and skin hydration measurement. Upon meeting the selection criteria, 15 subjects per group were enrolled in the clinical trial through randomization based on gender and age using the SPSS statistical program. The selection criteria included a crow’s feet wrinkle scale (Supplementary Table S2) of grade 3 or higher and 49 arbitrary units (A.U.) of moisture in the cheeks as measured with a Corneometer CM825 (Courage&Khazaka electronic GmbH, Koln, Germany). Prior to the clinical study, the purpose, protocol, and risks of the experiment were thoroughly explained to the subjects, and informed consent was obtained. This clinical trial was designed as a randomized, double-blind, placebo-controlled, parallel trial (Figure 2).

Subjects assigned to the caviar, combination, or placebo groups consumed the sample or placebo supplement daily for eight weeks. At 0, 4, and 8 weeks, measurements of skin hydration, wrinkles, and elasticity were conducted. Unless otherwise noted, each subject washed their face, waited for 30 min under constant temperature and humidity (22–24 °C, 45–55%), and limited water intake for 1 h before skin analysis. Measurements were taken at the right-angle intersection between the corner of the eye and the tip of the nose, and the average of three measurements was calculated.

### 2.5. Skin Hydration

Skin hydration was measured at 0, 4, and 8 weeks using the Corneometer CM825 (Courage&Khazaka electronic GmbH). The Corneometer numerically displays skin capacitance at 30-40 μm below the stratum corneum, indicating hydration through the insulating ions remaining in the probe head (A.U.). Dermal hydration was measured using Moisturemeter D Compact (Delfin Technologies Ltd., Kuopio, Finland), which utilizes a 265 MHz high-frequency electromagnetic field to penetrate to a depth of 2 to 2.5 mm in the skin, measuring the tissue dielectric constant in terms of moisture content (%).

### 2.6. Skin Elasticity

Skin elasticity was measured visits using the Cutometer^®^ MPA 580 (Courage&Khazaka electronic GmbH, Koln, Germany) device. Real-time measurements of several parameters (Ua, Uf, Ur, Ue) were taken at a constant sound pressure of 450 mbar, with on-time 2.0 s and off-time 2.0 s. Parameters included Ue (immediate distension), Uv (delayed distension), Uf (final deformation), Ur (immediate retraction), and Ua (total recovery). Measurements of R2 (Ua/Uf, total elasticity), R5 (Ur/Ue, net elasticity), and R7 (Ur/Uf, ratio of elastic recovery to total deformation) were performed at the right-angle intersection of the corner of the eye and the tip of the nose.

### 2.7. Transepidermal Water Loss (TEWL)

TEWL confirmed water loss via sweating and evaporation on the epidermis through passive diffusion processes in the stratum corneum. Limit water intake 1 h before measuring TEWL and measure it using a Vapometer^®^ (Delfin Technologies Ltd., Kuopio, Finland). Measure the right-angle intersection between the corner of the eye and the tip of the nose and use the average value of three measurements.

### 2.8. Skin Melanin

At 0, 4, and 8 weeks, the skin’s melanin content was measured using Mexameter^®^ MX 18 (Courage&Khazaka electronic GmbH, Koln, Germany). The device displays the melanin index by digitizing the skin absorption rate in each wavelength region. Measure the right-angle intersection between the corner of the eye and the tip of the nose and use the average value of three measurements.

### 2.9. Skin Wrinkles

We took high-resolution photos through a Mark-Vu (PSIPLUS, Suwon-si, Republic of Korea) 18-megapixel lens. PRIMOSCR (GFM, Teltow, Germany), a three-dimensional wrinkle measuring device using Digital Micro Mirror Device (DMD) technology, measured crow’s feet using parameter values of Ra (arithmetic average of profile peak height within the total measurement length), Rmax (maximum peak-to-valley value Rt over the assessment length), and Rz (average maximum height of the profile). 

### 2.10. Safety Test and Adverse Reaction Test

Safety was assessed by monitoring all adverse events and results from hematological tests, blood chemistry tests, and urine tests. Hematological tests measured WBC, RBC, hemoglobin, hematocrit, and platelets. Blood chemistry tests included renal and liver function indicators such as ALP, γ-GT, AST, and ALT activity, total protein, BUN, and creatine kinase concentrations, total cholesterol, and triglycerides. Indicators of renal function, albumin, total cholesterol, triglycerides, glucose, and urine tests were performed. Urine tests measured bilirubin, urobilinogen, and nitrite.

### 2.11. Statistical Analysis

Statistical analyses were performed using IBM SPSS statistics 25.0. Before and after comparisons within groups were performed using Wilcoxon signed rank test. For comparison between groups, analysis of variance was performed with the Kruskal-Wallis test, followed by a post-hoc Mann-Whitney test. All analyses were performed at a significance value of 0.05.

## 3. Results

### 3.1. Free Amino Acid Contents and Peptide of CV

The free amino acid content for caviar and enzyme-treated caviar is presented in Table 3. The measured free amino acids were glycine, alanine, serine, threonine, cysteine, valine, leucine, isoleucine, methionine, proline, phenylalanine, tyrosine, tryptophan, aspartic acid, glutamic acid, asparagine, glutamine, histidine, lysine, and arginine. Before enzyme treatment, the content of each free amino acid in the supercritical extract caviar powder is 0.73, 0.33, 0.15, 0.33, 0.4, 0.28, 0.14, 0.11, 0.34, 0.23, 0.17, 0.3, 0.05, 0.31, 0.11, 0.07, 0.23, and 0 mg/g, respectively. After enzyme treatment, the content of each free amino acid in CV is 10.32, 3.63, 5.78, 2.49, 3.49, 1.86, 1.51, 2.18, 3.6, 1.67, 1.34, 1.9, 0.59, 0.53, 0.86, 0.42, 0.66, and 0.41 mg/g, respectively. The content of free amino acids in CV was highest in the order of leucine, tyrosine, phenylalanine, and valine. 

**Figure 3:**
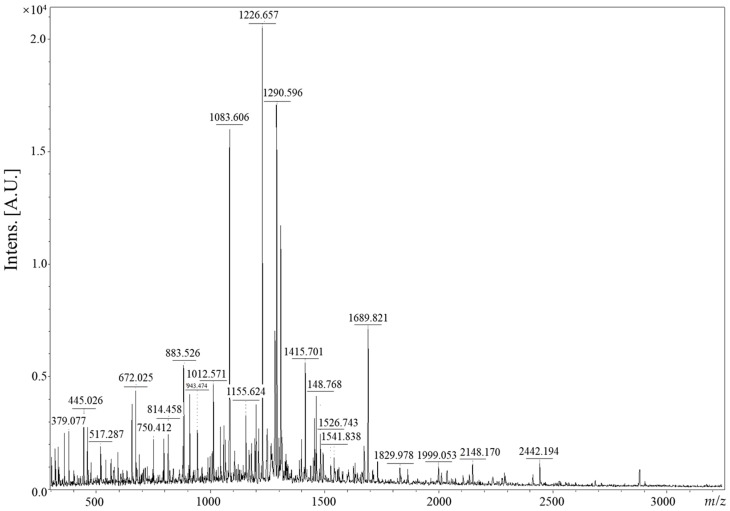
MALDI-TOF mass spectra of enzyme-treated caviar extract.

The peptide molecular weights of CV were determined using MALDI-TOF mass spectrometer (Bruker, Billerica, MA, USA) and are presented in Figure 3. The analysis revealed prominent CV peptides at 1083.606, 1226.657, and 1290.056 *m*/*z* (mass-to-charge ratio). A total of five peptides, each exhibiting an intensity (A.U.) of 0.5 or higher, are distributefigured across the mass-to-charge ratio range of 883.526 to 1689.821 *m*/*z*. Furthermore, the predominant constituents consist of peptides characterized by relatively low molecular weights, primarily falling within the range of 0 to 2000 *m*/*z*.

### 3.2. Effects of CV on Skin Hydration

After eight weeks of sample capsule intake, skin hydration changed from 43.091 ± 5.209 A.U. to 45.980 ± 5.008 A.U. in the caviar group, from 46.398 ± 1.484 A.U. to 47.438 ± 1.433 A.U. in the combination group, and from 44.940 ± 2.631 A.U. to 45.513 ± 2.514 A.U. in the placebo group. A significant difference was observed between the caviar and combination groups before and after sample intake, with both groups showing a significant difference compared to the placebo group.

**Figure 4:**
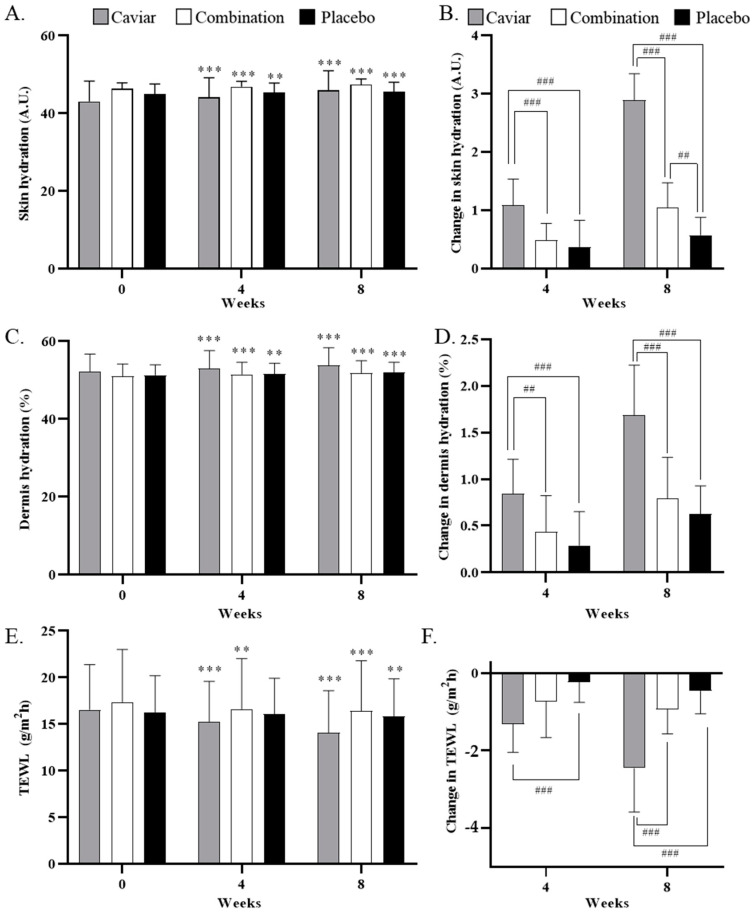
Effects of CV on skin hydration, dermal hydration, and change of hydration. (**A**) Measurement of skin hydration, (**B**) change of skin hydration, (**C**) measurement of dermal hydration, (**D**) change of dermal hydration (**E**) TEWL value and (**F**) change of TEWL in caviar, combination and placebo group at 4 and 8 weeks. * Compared within a group (** *p* < 0.01 and *** *p* < 0.001) with Wilcoxon signed rank test. ^#^ compared to placebo group (^##^ *p* < 0.01 and ^###^ *p* < 0.001) with Mann–Whitney test.

Dermal hydration after eight weeks changed from 52.156 ± 4.580 A.U. to 53.844 ± 4.538 A.U. in the caviar group, from 52.156 ± 4.580 A.U. to 53.844 ± 4.538 A.U. in the combination group, and from 51.069 ± 3.105 A.U. to 51.860 ± 3.189 A.U. in the placebo group. A significant difference was noted between the two experimental groups before and after sample intake, but only the caviar group exhibited a significant difference compared to the placebo group after treatment (Figure 4C,D). 

The effects of CV on TEWL are depicted in Figure 4E,F. After eight weeks of sample intake, TEWL in the combination group decreased from 17.356 ± 5.653 to 16.416 ± 5.390, the caviar group decreased from 16.551 ± 4.834 to 14.096 ± 4.489, and the placebo group decreased from 16.289 ± 3.917 to 15.827 ± 4.040. TEWL significantly improved in both treatment groups after eight weeks.

### 3.3. Skin Elasticity

**Figure 5:**
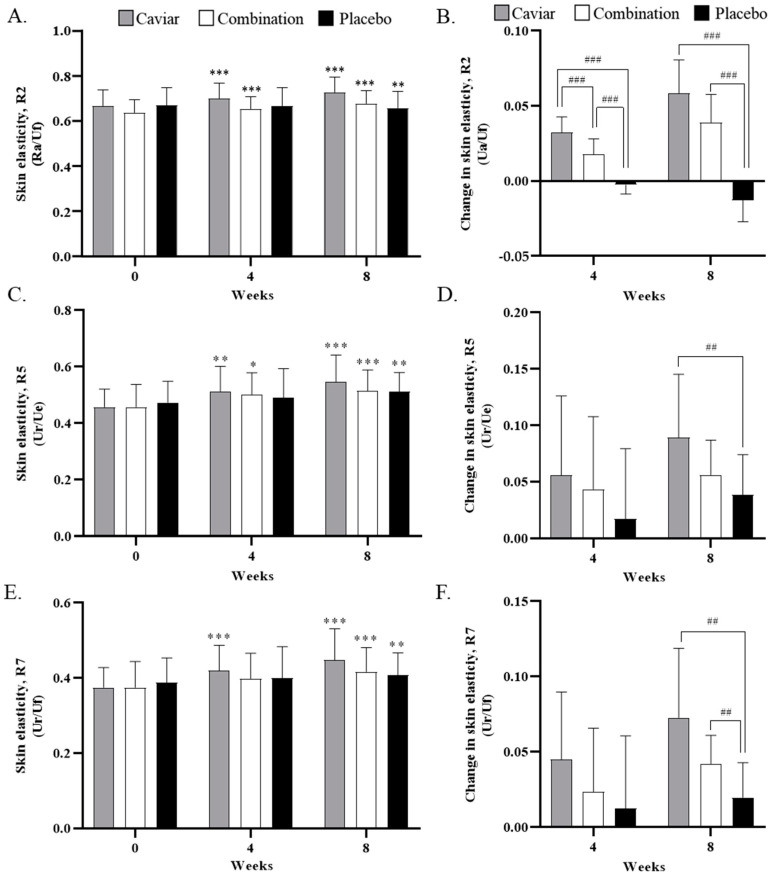
Effects of CV on skin elasticity. (**A**) R2, overall elasticity, (**B**) change rate of skin elasticity (R2), (**C**) R5, the net elasticity, (**D**) change rate of skin elasticity (R5), (**E**) R7, elastic recovery to total deformation, (**F**) change rate of skin elasticity (R7) with administration of caviar, combination, or placebo for 4 weeks and 8 weeks. * Compared within a group (* *p* < 0.05, ** *p* < 0.01 and *** *p* < 0.001) with Wilcoxon signed rank test. ^#^ compared to placebo group (^##^ *p* < 0.01 and ^###^ *p* < 0.001) with Mann–Whitney test.

Results for skin elasticity are illustrated in Figure 5. After eight weeks of sample intake, the R2 value in the placebo group decreased from 0.668 ± 0.080 to 0.655 ± 0.076, the combination group increased from 0.637 ± 0.056 to 0.676 ± 0.058, and the caviar group increased from 0.667 ± 0.070 to 0.726 ± 0.069. The R2 value significantly increased in both experimental groups and decreased in the control group. The change in R2 value was significantly different in both the caviar and combination groups compared to the placebo group at eight weeks (Figure 5A,B).

After eight weeks of sample intake, the R5 value in the placebo group decreased from 0.474 ± 0.075 to 0.512 ± 0.068, while in the caviar and combination groups, it increased from 0.457 ± 0.064 to 0.547 ± 0.095 and from 0.458 ± 0.080 to 0.514 ± 0.075, respectively. Caviar showed a significant increase at eight weeks compared to the placebo group (Figure 5C,D). 

After eight weeks of sample intake, the R7 value in the placebo group decreased from 0.387 ± 0.066 to 0.407 ± 0.060, the combination group increased from 0.375 ± 0.069 to 0.417 ± 0.064, and the caviar group increased from 0.375 ± 0.053 to 0.447 ± 0.084. After eight weeks, the R7 value significantly improved in all groups, and the change in R7 value significantly increased in both treatment groups compared to the placebo group (Figure 5E,F).

### 3.4. Effects of CV on Skin Melanin Index and Wrinkles

**Figure 6:**
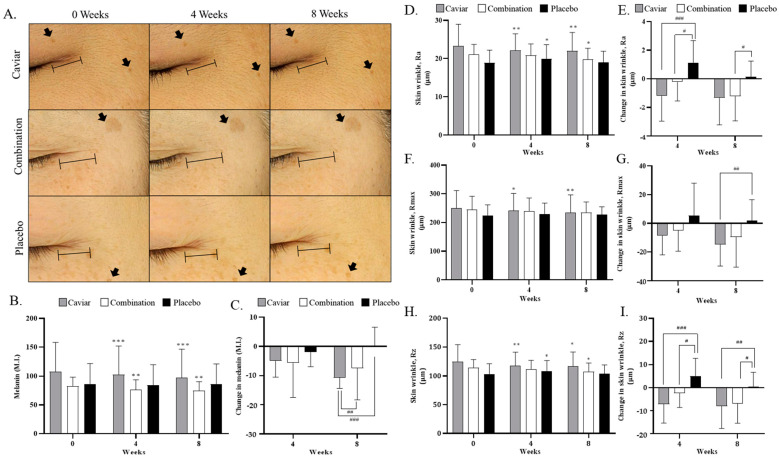
Effects of CV on Skin wrinkles. (**A**) High-resolution skin photos using Mark-Vu (**B**) Melanin index and (**C**) change of melanin index (**D**) Ra value, average roughness, (**E**) change in skin wrinkle Ra (**F**) Rmax value, maximum peak-to-valley distance, (**G**) change in skin wrinkle Rmax, (**H**) Rz value, average maximum height of the profile and (**I**) change in skin wrinkle Rz of the caviar, combination, and placebo group for 4 and 8 weeks. Bar represents skin wrinkles, and arrows represent liver spots. * Compared within a group (* *p* < 0.05, ** *p* < 0.01 and *** *p* < 0.001) with Wilcoxon signed rank test. ^#^ compared to placebo group (^#^ *p* < 0.05, ^##^ *p* < 0.01 and ^###^ *p* < 0.001) with Mann–Whitney test.

To assess the whitening effect of CV, the melanin index measurement results are shown in Figure 6. The melanin index of the combination group decreased from 80.222 ± 16.655 M.I. to 74.756 ± 15.580 M.I., that of the caviar group decreased from 107.711 ± 50.620 M.I. to 96.978 ± 49.545 M.I., and that of the control group changed from 86.378 ± 35.379 to 86.444 ± 34.876 M.I. after eight weeks of sample intake (Figure 6B,C). Changes in the melanin index were significantly decreased in both the caviar and combination groups compared to the placebo group at eight weeks.

Wrinkles were measured using PRIMOSCR (GFM, Teltow, Germany) at the right-angle intersection of the nose and the tip of the eye. Wrinkle parameters included Ra (average roughness), Rmax (maximum peak to valley distance over the assessment length), and Rz (average maximum profile height). After eight weeks of sample intake, the Ra value in the combination group decreased from 21.093 ± 2.620 μm to 19.869 ± 2.851 μm, that of the caviar group decreased from 23.347 ± 5.647 μm to 22.007 ± 4.823 μm, and that of the placebo group decreased from 18.862 ± 3.360 μm to 19.005 ± 2.934 μm. The change in Ra value was significantly improved in both treatment groups (Figure 6D,E).

The Rmax value decreased from 244.442 ± 46.975 μm to 234.957 ± 36.421 μm in the combination group and from 249.927 ± 61.225 μm to 235.144 ± 61.048 μm in the caviar group, while it increased from 224.223 ± 36.487 μm to 226.752 ± 27.608 μm in the placebo group. There was a significant improvement only in the caviar group compared to the placebo group (Figure 6F,G).

The Rz value of the combination group decreased from 114.493 ± 14.097 μm to 107.556 ± 15.006 μm, that of the caviar group decreased from 124.783 ± 29.388 μm to 116.770 ± 24.634 μm, and that of the placebo group increased from 103.663 ± 17.749 μm to 104.239 ± 14.970 μm. The change in Rz value was significantly improved in both treatment groups compared to the placebo group (Figure 6H,I).

### 3.5. Safety and Adverse Reaction Evaluation

Table 4 presents the analysis results of the safety and adverse reaction evaluation of this study. Diagnostic test items were measured before and eight weeks after sample intake. Among the hematological tests, there were significant intra-group differences in monocytes and eosinophils in the caviar group, and significant differences between groups in Hb and Hct. However, the investigator determined them to be of no clinical significance. There was no statistically significant difference among the three groups after eight weeks of sample intake in all parameters of the biochemical test. Similarly, there was no statistically significant difference among the three groups after eight weeks of ingestion in all items of the biochemical/urine test. There were no reports of serious adverse events while the subjects participated in the trial.

## 4. Discussion

Caviar, internationally acclaimed as a highly esteemed seafood, is prized for its abundance of protein and fat, establishing it as an exceptional source of high-quality protein in food [19,20]. Cosmetics containing caviar extract have been reported to improve the appearance of photoaged skin. Additionally, the caviar extract has demonstrated benefits in wound healing, anti-inflammatory responses, and collagen synthesis [4,5]. In a preceding study, we established the skin anti-aging efficacy of enzyme-treated caviar extract (CV) through antioxidant and anti-inflammatory effects in UVB-irradiated hairless mice and HaCaT cells (Figure 1) [15]. To ascertain the applicability of these results in humans, we evaluated the skin anti-aging efficacy of CV intake in a clinical trial. Additionally, we confirmed the effects of enzyme treatment of caviar extract on free amino acid composition and peptide molecular weight. This is the first report on the anti-aging efficacy of caviar in human skin for use as nutraceuticals.

Previous studies have demonstrated that the protein, carbohydrate, and ASH content of caviar increased more in supercritical CO_2_ extract than in 70% ethanol extract. In this experiment, we confirmed the effects of enzyme treatment on the amino acid content of supercritical CO_2_ extract [15]. Protamex enzyme used in this experiment is an exopeptidase, and it hydrolyzes the protein that was first decomposed by Prozyme1000L to a smaller unit by performing the second hydrolysis. The free amino acid content was higher in caviar enzyme-treated powder than in general supercritical extract residue (Table 3). The caviar extract was treated with enzymes to increase the free amino acid content by more than 10 times. Similar to other caviar, it is mainly composed of leucine, tyrosine, and phenylalanine [21]. Furthermore, the CV predominantly comprises peptides with a molecular weight of approximately 1200 *m*/*z*, suggesting the presence of low-molecular-weight peptides composed of approximately 10 amino acids or fewer in CV. Ingestion of these free amino acids can be absorbed by the body faster than when ingesting protein. Recent clinical trials have shown that the plasma amino acid concentration in the free amino acid intake group was higher than that in the protein intake group, and when analyzing the amount of phenylalanine in plasma 6 h after a meal, it was higher in the free amino acid intake group [22]. This indicates that ingestion of free amino acids has a faster absorption rate than ingestion of protein form and results in high availability in the body. In conclusion, we concluded that CV exhibits more effective skin anti-aging effects than general caviar extract because of its predominant composition of low-molecular-weight peptides containing leucine, tyrosine, and phenylalanine.

In the clinical trial, a caviar group was formulated to assess the effects of caviar extract alone, while a combination group was included as an example of a finished product, containing well-known ingredients recognized for their positive impact on skin elasticity, hydration, and wrinkle improvement, such as fish collagen, hyaluronic acid, and ascorbic acid [23,24]. To evaluate efficacy, we first investigated the skin hydration effect in humans who administered caviar and its combination ingredients once a day for eight weeks. Both the caviar and combination groups exhibited a significant increase in skin hydration, dermal hydration, and TEWL levels compared to the placebo group. Notably, the caviar group displayed over three times the improvement in dermis and endothelium hydration compared to the placebo group. ROS reduction in hyaluronan synthesis or decomposition of hyaluronan molecules contributes to dry skin [25,26]. Our study revealed that CV increased the production of sphingomyelin and hyaluronic acid through heightened mRNA expression levels of HAS1, HAS2, and HAS3 in UVB-irradiated mice [15] (Figure 1). Thus, the observed increase in skin moisture following CV administration in clinical trials suggests its regulatory impact on hyaluronan synthase.

Type 1 collagen induces collagen synthesis in human dermal fibroblasts, while MMPs degrade collagen [27]. Collagen deficiency results in wrinkles and loss of elasticity [28,29,30]. Caviar extract administration significantly improved skin wrinkles (Ra, Rmax, Rz) and elasticity (R2, R5, R7) compared to the placebo group. In vivo studies indicated a significant increase in the expression of pro-collagen type I and collagen type I, along with a significant decrease in the expression of c-FOS, c-JUN, and MMP-1, -3, -9, etc. (Figure 1). The observed improvement in skin wrinkles and elasticity with CV is attributed to increased collagen synthesis and decreased collagen degradation. The data showed no significant differences in Rp (maximum profile peak height) and Rv (maximum profile valley depth) between groups, but there were significant differences in all groups before and after sample intake. CV extract affected Ra, Rmax, and Rz, but not Rv and Rp. Thus, it was confirmed that caviar extract had no significant effect on each maximum profile peak height and valley depth but affected the overall average and sum.

UVB radiation induces ROS, causing DNA damage, premature senescence, and melanin production within melanocytes, along with tyrosinase (TYR) [31]. CV demonstrated whitening effects by down-regulating PKA/CREB/MITF/TRP in the melanogenesis pathway (Figure 1) [16]. CV administration significantly reduced melanin levels in group caviar and combination group, showcasing its effectiveness in increasing elasticity and reducing melanin levels. Overall experiments revealed that both the caviar and combination groups displayed high skin anti-aging efficacy, encompassing skin hydration, whitening, wrinkles, etc., with the effect of caviar generally surpassing that of the combination. Therefore, it is inferred that higher caviar extract content correlates with better skin anti-aging efficacy.

Clinical pathology tests, including hematological, biochemical, and urine tests, were conducted to ensure the safety of CV. To ensure safety, clinical pathology tests such as blood tests, biochemical tests, and urine tests were conducted. Safety evaluations revealed that almost all values exhibited no significant differences within or between groups. Some clinical hematological test results or biochemical test items showed significant differences between groups; however, these variations were within the clinical laboratory reference values, making it challenging to assign clinical significance. Based on the confirmed data, CV can be considered an effective food ingredient for improving skin conditions.

## 5. Conclusions

In conclusion, this study unequivocally established the skin anti-aging efficacy and safety of CV through a randomized, placebo-controlled, and double-blind clinical trial. Over an 8-week period, CV administration resulted in a significant improvement in skin parameters, including hydration, elasticity, whitening, and wrinkles, when compared to the placebo group. Notably, there were no clinically significant adverse reactions or noteworthy findings in the safety evaluation factors observed. As a result, CV improves factors such as skin hydration, elasticity, and wrinkles due to high free amino acids and low molecular weight peptides. This study demonstrates caviar’s potential as a functional food for skin anti-aging.

## Figures and Tables

**Table 1:** Composition of daily dose (900 mg) capsule.

Composotion	Caviar Group	Combination Group	Placebo Group
Enzyme-Treated Caviar Extract	500 mg	10 mg	
Maltodextrin	386 mg		886 mg
Silicon dioxide	14 mg	12 mg	14 mg
Fish Collagen		500 mg	
Hyaluronic acid		240 mg	
Ascorbic acid		125 mg	
Biotin		7.5 mg	
d-α-tocopherol		5.5 mg	

**Table 2:** Demographics and baseline characteristics.

Characteristics		Caviar Group	Combination Group	Placebo Group
Age		47.87 ± 8.27	48.07 ± 8.25	48.07 ± 6.84
Sex	Male	3	1	2
	Female	12	14	13
Skin wrinkles (grade)		4.13 ± 0.83	3.93 ± 1.10	4.267 ± 1.03

**Table 3:** Free amino acid contents in supercritical extract and enzyme-treated supercritical caviar extract.

Free Amino Acid	Supercritical CO_2_ Caviar Extract (mg/g)	Enzyme-Treated Supercritical Caviar Extract (mg/g)
Leucine	0.73	10.32
Phenylalanine	0.33	3.63
Tyrosine	0.15	5.78
Alanine	0.33	2.49
Isoleucine	0.4	3.49
Valine	0.28	1.86
Arginine	0.14	1.51
Methionine	0.11	2.18
Lysine	0.34	3.6
Serine	0.23	1.67
Threonine	0.17	1.34
Glutamic acid	0.3	1.9
Histidine	0.05	0.59
Aspartic acid	0.31	0.53
Threonine	0.11	0.86
Glycine	0.07	0.42
Proline	0.23	0.66
Cystine	-	0.41

**Table 4:** Safety outcome measures between treatment group and placebo group at 0 weeks and after 8 weeks intake caviar, combination, and placebo.

Parameter	Caviar Group		Combination Group		Placebo Group	
Hematological test ^(1)^	0 weeks	8 weeks	0 weeks	8 weeks	0 weeks	8 weeks
WBC (10^9^/L)	5.61 ± 1.67	5.40 ± 1.340	4.75 ± 1.24	4.26 ± 1.35	5.24 ± 1.20	4.70 ± 1.23
RBC (10^12^/L)	4.63 ± 0.46	4.54 ± 0.43	4.37 ± 0.40	4.30 ± 0.32	4.24 ± 0.32	4.19 ± 0.25
Hb (g/dL)	13.78 ± 1.78 ^#^	13.45 ± 1.65 *	11.91 ± 2.52	11.64 ± 2.39	12.61 ± 1.60	12.27 ± 1.40
Hct (%)	42.83 ± 4.63 ^#^	41.49 ± 4.32	38.30 ± 6.19	37.30 ± 5.71	39.31 ± 3.93	38.35 ± 3.31
Platelet (10^9^/L)	250.67 ± 55.39	258.80 ± 55.30	280.80 ± 58.75	280.53 ± 49.41	279.20 ± 51.99	268.40 ± 57.70
Seg. Neutrophil (%)	55.27 ± 9.46	52.19 ± 8.77	55.07 ± 8.91	51.99 ± 8.64	52.95 ± 8.18	50.79 ± 9.18
Lymphocyte (%)	35.49 ± 8.70	37.00 ± 7.95	35.97 ± 8.67	37.55 ± 7.70	36.77 ± 7.30	38.40 ± 8.44
Monocyte (%)	6.68 ± 1.28	7.45 ± 1.50 *	6.51 ± 1.48	7.14 ± 1.78	6.98 ± 1.58	7.64 ± 2.09
Eosinophil (%)	1.87 ± 0.87	2.59 ± 1.41 *	1.64 ± 0.83	2.41 ± 2.37	2.41 ± 2.11	2.41 ± 1.56
Basophil (%)	0.69 ± 0.24	0.73 ± 0.33	0.81 ± 0.49	0.91 ± 0.51	0.89 ± 0.40	0.77 ± 0.43
Biochemical test ^(2)^						
Glucose (mg/dL)	91.20 ± 12.57	92.07 ± 8.88	89.93 ± 5.42	88.67 ± 7.07	88.67 ± 7.07	88.47 ± 7.74
T.Protein (g/dL)	6.96 ± 0.40	7.03 ± 0.37	6.86 ± 0.37 *	6.97 ± 0.41 *	6.85 ± 0.50	6.90 ± 0.42
Albumin (mg/dL)	0.67 ± 0.19	0.63 ± 0.24	0.70 ± 0.33	0.73 ± 0.34	0.65 ± 0.16	0.69 ± 0.15
BUN (mg/dL)	12.40 ± 3.29	13.47 ± 3.58	12.07 ± 2.94	12.00 ± 2.98	13.67 ± 3.98	14.07 ± 5.18
AST (U/L)	22.00 ± 7.41	21.40 ± 6.13	19.87 ± 10.11	19.67 ± 7.00	20.60 ± 4.93	20.80 ± 4.86
ALT (U/L)	25.14 ± 15.98	23.20 ± 16.01	17.36 ± 13.07	18.39 ± 10.81	13.93 ± 5.86	15.13 ± 4.78
γ-GTP (U/L)	25.27 ± 12.93	23.33 ± 11.48	23.33 ± 25.21	19.00 ± 8.61	18.47 ± 7.31	18.00 ± 8.34
Creatinine (mg/dL)	0.68 ± 0.15	0.67 ± 0.15	0.64 ± 0.13	0.63 ± 0.12	0.67 ± 0.16	0.66 ± 0.123
Total Cholesterol (mg/dL)	197.40 ± 31.30	203.27 ± 32.13	191.20 ± 34.55	193.20 ± 34.97	200.40 ± 34.77	194.13 ± 33.12
HDL-Cholesterol (mg/dL)	61.07 ± 15.16	60.40 ± 16.49	61.07 ± 14.21	60.20 ± 13.36	64.07 ± 12.38	61.13 ± 11.63
LDL- Cholesterol (mg/dL)	119.13 ± 22.06	121.47 ± 25.07	111.00 ± 29.81	108.73 ± 28.78	119.33 ± 29.04	113.47 ± 27.90
Triglyceride (mg/dL)	92.53 ± 59.70	89.27 ± 40.26	87.00 ± 51.61	102.00 ± 69.71	79.60 ± 27.09	77.80 ± 28.08
Urine test (abnormal personnel)						
Specific gravity	0	0	0	0	0	0
pH	2	0	2	0	1	0
Protein	1	0	2	2	1	2
Glucose	0	0	0	0	0	0
Ketone	0	0	0	0	0	0
Bilirubin	0	0	0	0	0	0
Urobilinogen	0	0	0	0	0	0
Nitrite	0	0	0	1	0	0
RBC	1	0	2	1	0	0
WBC	0	1	0	4	2	1
Number of adverse reactions (persons)	0	0	0	0	0	0

^(1)^ WBC: white blood cells, RBC: red blood cells, Hb: hemoglobin. ^(2)^ Hct: Hematocrit, BUN: blood urea nitrogen, AST: aminotransferase, ALT: alanine aminotransferase. * compared within a group (* *p* < 0.05) with Wilcoxon signed rank test. ^#^ compared to the placebo group (^#^ *p* < 0.05) with Mann–Whitney test.

## Data Availability

Data supporting the study results can be provided followed by request sent to the corresponding author’s e-mail.

## Supplementary Materials

The following supporting information can be downloaded at: https://www.mdpi.com/article/10.3390/nu16010137/s1, Table S1: CONSORT 2010 checklist of information to include when reporting a randomised trial; Table S2: Skin wrinkle visual evaluation.

## Abbreviations

SOD, Superoxide dismutase; GPx, glutathione peroxidase; MAPK, mitogen-activated protein kinase; JNK, c-Jun N-terminal kinase; NF-κB, nuclear factor-κB; JNK, Jun NH2-terminal kinas; TNF-α, tumor necrosis factor α; IL, interleukin; SPT, serine palmitoyltransferase; CerS, ceramide synthase; Des, dihydroceramide desaturases; TRP, tyrosinase-related protein; cAMP, cyclic adenosine monophosphate; PKA, cAMP-protein kinase A; MITF, microphthalmia-associated transcription factor; HAS, Hyaluronic Acid Synthases.
